# Knowledge and practice of COVID-19 prevention among community health workers in rural Cross River State, Nigeria: implications for disease control in Africa

**DOI:** 10.11604/pamj.2020.37.50.24307

**Published:** 2020-09-14

**Authors:** Ogban Omoronyia, Nnette Ekpenyong, Ikechukwu Ukweh, Enagu Mpama

**Affiliations:** 1 Department of Community Medicine, University of Calabar, Calabar, Nigeria

**Keywords:** COVID-19, knowledge, prevention, community health workers, Nigeria

## Abstract

**Introduction:**

in developing countries, community health workers (CHWs) are essential, for provision of behaviour change communication towards prevention of coronavirus 2019 (COVID-19) infection at rural grassroots level. We aimed at assessing their level of knowledge and practice of preventive measures in a developing country setting.

**Methods:**

total enumeration of all CHWs in a rural local government area in southern Nigeria was carried out, using cross-sectional descriptive study design. Pretested self-administered 15-item questionnaire was used to assess knowledge of COVID-19 including basic epidemiology, virology, preventive measures and use of personal protective equipment (PPE). Practice of preventive measures was also assessed. Each correct response to knowledge question contributed one unit to the total score which was converted to percentage. Total knowledge score of 50% or greater was considered satisfactory.

**Results:**

complete data was obtained from eighty-six (86) respondents with mean age of 36.3±8.9 years (18-54 years). Mean total knowledge score was 28.14±12.8% (6.7 to 53.3%), and 9.3% (n=8) had score of at least 50%. Correct responses to appropriate sequence of putting on and removing personal protective equipment (PPE) were 5.8% (n=5) and 8.1% (n=7), respectively. Regular practice of use of face masks, goggles, gloves, and hand hygiene was found to be 50% (n=43), 12.8% (n=11), 30.2% (n=26), and 56.4% (n=48), respectively.

**Conclusion:**

community health workers are grossly underprepared for provision of health education on COVID-19, due to their poor level of knowledge. Their capacity building through workshops and effective continuing education program are urgently needed.

## Introduction

Among many emerging and re-emerging infectious diseases that have plagued man, the recent coronavirus 2019 (COVID-19) infection has perhaps made the most far-reaching global impact [1]. Most of the entire world has remained in lock-down following the pandemic onset in Wuhan, China, and subsequent global spread in early 2020 [2]. Despite its devastating effect on healthcare system with over 400,000 deaths reported so far, there is yet no definite cure or vaccine [3]. Infection with the novel virus which usually presents with fever, cough and difficulty breathing, is therefore mostly managed symptomatically, with prevention as key thrust for control of its spread [1, 3]. Unfortunately, many healthcare systems are already getting overwhelmed, with increasing paucity of isolation centres, treatment facilities and trained personnel [4]. Unlike other regions and continents of the world, sub-Saharan Africa (SSA) has had much fewer cases, as well as reported much less mortality from the pandemic [3]. However, while there appears to be a plateauing or slowing of the trend in much of the Western world, many countries in SSA are experiencing resurgence and rapid rise in new cases [1, 3]. This trend may be worse, especially with poor planning of ease of lock down and resumption of social and other activities in densely populated countries, including Nigeria [5]. Increased risk of disease spread may have higher impact on at-risk groups including healthcare workers, who are at the frontline in control of the pandemic [5-7]. Their practice of preventive measures is therefore key to prevention of loss of skilled personnel, who are essential to sustenance of prevention effort [7].

In many developing countries, rural settlements constitute larger proportion of the population compared with urban cities [8]. Due to lack of skilled health personnel in most rural health facilities, community health workers are often left as the frontline personnel with the most direct and frequent contact with grassroots community members [9]. They are key to dissemination of information required for adoption of appropriate preventive behaviour by the vast majority of the population. Many developing country health care systems may therefore have to depend on CHOs for control of disease spread [9]. However, considering the novel nature of COVID-19 and the curriculum of training of CHOs, their capacity as front liners in the practice of preventive measures, as well as dissemination of health information may be in doubt [10]. This study was therefore aimed at assessing level of knowledge and practice of COVID-19 preventive measures among CHOs comprising community health extension workers (CHEWs) and community health officers (CHOs) in rural settlements in Cross River State, southern Nigeria.

## Methods

**Study design and population:** this study was conducted using cross-sectional descriptive design. Study area was Yala Local Government Area (LGA) in northern senatorial district of Cross River State, southern Nigeria. The LGA comprised fourteen (14) wards and eighty one (81) health facilities. Study population was community health workers comprising community health extension workers (CHEW) and community health officers (CHO). This globally recognized group of healthcare professional, were established and technically designed to reach out to, and provide basic preventive health services to community members at grassroots level [11].

**Sampling and instrument:** total enumeration was carried out, with recruitment of all CHWs from these facilities in all but one of these wards, which was excluded due to on-going communal clashes during the study period. Self-administered questionnaire was used to obtain quantitative data on knowledge and practice of COVID-19 prevention. The structured questionnaire comprised three sections. Section 1 assessed sociodemographic and occupational characteristics of respondents. Section 2 comprised 15-items which assessed knowledge of COVID-19 epidemiology, virology, preventive measures and use of personal protective equipment (PPE). Section 3 assessed practice of preventive measures, including use of face mask, gloves, and hand hygiene. Responses given as ‘Always’ and ‘Almost always’ were considered regular practice, while ‘Sometimes’, ‘Rarely’ and ‘Never’, were considered not regular practice of preventive measures. Pretesting of the questionnaire was conducted among CHWs in similar socio-demographic setting, with Crombach's alpha of 0.83 before use for data collection.

**Data analysis:** data entry and analysis were done using SPSS version 21.0. Each correct response to knowledge question contributed one unit to the total score which was converted to percentage. A score of 50% or greater was considered satisfactory for this cadre of health professionals. Practice of each of the preventive measures was graded as regular for those with always and almost always, and non-regular for those with sometimes, occasional or rarely. Independent t-test was used as inferential statistics to assess factors associated with regular practice of these measures. P-value was set at 0.05.

**Ethical considerations:** ethical approval to conduct the study was obtained from the Research Ethics Committee of Cross River State Ministry of Health, Calabar. Written consent was obtained from respondents before data collection.

## Results

**Sociodemographic characteristics:** complete data was obtained from eighty-six respondents with response rate of 100% and female: male ratio of 1: 0.62. Mean age was 36.3±8.9 years (18-54 years). Most subjects were within 21-40 years old, CHEW, and had worked for 10 years or less, comprising 68.6% (n=59), 82.6% (n=71), and 59.3% (n=51), respectively (Table 1).

**Table 1:** occupational characteristics of respondents (N=86)

Variable	Frequency	Percentage
**Gender**		
Male	33	38.4
Female	53	61.6
Total	86	100
**Age group (in years)**		
<20	4	4.7
21-30	18	20.9
31-40	41	47.7
41-50	16	18.6
>50	7	8.1
Total	86	100
**Cadre of healthcare worker**		
CHEW^*^	71	82.6
CHO^**^	15	17.4
Total	86	100
**Duration of practice (in years)**		
<10	51	59.3
11-20	25	29.1
>20	10	11.6
Total	86	100

* CHEW=Community Health Extension Worker; ^**^CHO=Community Health Officer

**Knowledge of COVID-19:** Table 2 shows frequency distribution of correct responses to each of the questions in each of the sections of knowledge of COVID-19. Overall, questions with relatively higher frequency of correct responses were main mode of transmission and method of COVID-19 prevention in hospital setting, comprising 67.4% (n=58) and 61.6% (n=53), respectively. Questions with lower frequency of correct response were correct sequence of putting on, and removing personal protective equipment (PPE), comprising 5.8% (n=5) and 8.1% (n=7), respectively. Mean total knowledge score was 28.14±12.8% ranging from 6.7% to 53.3%. Most respondents comprising 80.2% (n=69), had scores within 10% to 39% (Table 3). Mean scores for knowledge of basic virology and epidemiology, knowledge of COVID-19 prevention, and knowledge of use of PPE were 38.8±18.5%, 25.8±18.6% and 19.8±9.9, respectively. Less than one-tenth of respondents comprising 9.3% (n=8), had total knowledge score of at least 50%. Most subjects comprising 66.2% (n=57), 61.6% (n=53) and 74.4% (n=64), had scores within 40-60% for knowledge of basic virology and epidemiology, 20% or less for both knowledge of COVID-19 prevention, and use of PPE, respectively.

**Table 2:** frequency distribution of correct responses questions on knowledge of COVID-19 (N=86)

S/N	Knowledge question	Frequency of correct response	Percentage of correct response
**Basic virology and epidemiology of COVID-19**			
1	The virus causing COVID-19 infection is called	33	38.4
2	The main mode of transmission of virus from person to person is via:	58	67.4
3	Which of the following is considered a “close contact”?	22	25.6
4	Which of the following individuals are generally at higher risk of COVID-19 infection	17	19.8
5	Which of the following hand hygiene practices does not prevent transmission of the virus to a health worker?	37	43.0
**Basic knowledge of preventive measures**			
6	Which of the following is a medical advice indicated?	25	29.1
7	Preferred method of hand hygiene for visibly soiled hands is	11	12.8
8	Which of the following is the most effective method for prevention of COVID-19 infection in the healthcare setting?	53	61.6
9	Which of the following is recommended for isolation of a patient confirmed with, suspected, or under investigation for COVID-19 infection?	10	11.6
10	Which of the following are recommended infection control measures upon arrival if a patient suspected of having COVID-19 infection?	12	14.0
**Knowledge of use of PPE and COVID-19 management**			
11	What personal protective equipment may not be worn by individuals transporting patients who are confirmed with, suspected, or under investigation for COVID-19 infection?	31	36.0
12	Which of the following is the correct sequence for putting on PPE?	5	5.8
13	Which of the following is the correct sequence for removing PPE?	7	8.1
14	Which of the following is incorrect concerning removal of PPE?	23	26.7
15	Which of the following is/are currently approved standard treatment for COVID-19 infection?	19	22.1

**Table 3:** distribution of knowledge scores by knowledge area (N=86)

Knowledge area	Frequency	Percentage
**Total knowledge score percent**		
<10%	6	7.0
10-19%	29	33.7
20-29%	18	20.9
30-39%	22	25.6
40-49%	3	3.5
>50%	8	9.3
Total	86	100.0
**Basic virology and epidemiology**		
0%	5	5.8
20%	22	25.6
40%	34	39.5
60%	23	26.7
80%	2	2.3
Total	86	100.0
**Basic knowledge on COVID-19 prevention**		
0%	18	20.9
20%	35	40.7
40%	23	26.7
60%	10	11.6
Total	86	100.0
**Knowledge of use of PPE and COVID-19 treatment**		
0%	33	38.4
20%	31	36.0
40%	12	14.0
60%	10	11.6
Total	86	100.0

**Practice of preventive measures:** regular practice (comprising always and most times) of use of face mask, goggles, gloves, and hand hygiene was found to be 50% (n=43), 12.8% (n=11), 30.2% (n=26), and 56.4% (n=48), respectively (Table 4). Approximately two-thirds of respondents comprising 67.4% (n=58), had rarely or never used goggles or face shield when in contact with suspected cases of COVID-19 infection. Table 5 shows factors associated with practice of COVID-19 prevention measures. Age and duration of practice were not found to be associated with practice of each of the preventive measures. For each of the preventive measures, mean scores for total as well as each of the sections of knowledge, were found to be higher among respondents with regular compared with non-regular practice, though this differences were not statistically significant (p>0.05).

**Table 4:** practice of COVID-19 prevention measures by healthcare workers (N=86)

sn	Variable	Regular		Not regular		
		Always n (%)	Almost always n (%)	Sometime n (%)	Rarely n (%)	Never n (%)
1	How often do you wear or use face mask when in contact with any patient during this period of COVID-19 pandemic?	14 (16.3)	29 (33.7)	43 (50.0)	0 (0.0)	0 (0.0)
2	How often do you wear or use goggles or face shield when in contact with any patient during this period of COVID-19 pandemic?	0 (0.0)	11 (12.8)	17 (19.8)	22 (25.6)	36 (41.8)
3	How often do you wear or use gloves when in direct contact with any patient during this period of COVID-19 pandemic?	2 (2.3)	24 (27.9)	48 (55.8)	10 (11.7)	2 (2.3)
4	How often do you wash your hands with soap or use alcohol-based sanitizer after attending to any patient during this period of COVID-19 pandemic?	17 (20.0)	31 (36.4)	30 (35.3)	7 (8.1)	1 (1.2)

**Table 5:** factors associated with practice of COVID-19 preventive measures (N=86)

Variable	Use of face mask		Use of hand gloves		Hand washing	
	Regular	Not Regular	Regular	Not Regular	Regular	Not Regular
**Age** (years)						
Mean (SD)	36.9 (8.4)	35.7 (9.5)	37.9 (8.6)	35.7 (9.1)	37.0 (9.3)	35.5 (8.4)
t-test p-value	0.54		0.28		0.41	
**Duration of practice** (years)						
Mean (SD)	12.3 (7.8)	11.0 (7.6)	12.5 (7.6)	11.3 (7.8)	11.9 (8.2)	11.4 (7.1)
t-test p-value	0.43		0.51		0.78	
**Knowledge of epidemiology**						
Mean (SD) (%)	40.9 (6.7)	36.7 (10.0)	38.5 (12.1)	39.0 (10.4)	38.9 (8.5)	38.8 (8.6)
t-test p-value	0.3		0.9		0.96	
**Knowledge of basic prevention**						
Mean (SD) (%)	28.8 (8.7)	22.8 (8.3)	27.7 (8.8)	25.0 (8.6)	27.9 (8.3)	24.2 (8.9)
t-test p-value	0.13		0.54		0.36	
**Knowledge of use of PPE**						
Mean (SD) (%)	19.1 (7.3)	20.5 (9.7)	18.5 (9.5)	20.3 (10.3)	24.2 (12.6)	16.3 (10.4)
t-test p-value	0.75		0.69		0.07	
**Total knowledge score**						
Mean (SD) (%)	29.6 (10.2)	26.7 (11.3)	28.2 (5.4)	28.1 (4.6)	30.4 (8.5)	26.4 (9.9)
t-test p-value	0.29		0.98		0.16	

## Discussion

This study was aimed at assessing level of knowledge and practice of COVID-19 prevention among community health workers in a sub-Saharan African setting. This is perhaps one of few studies conducted among health workers that have frequent contact with community members in rural developing country settings [12, 13]. There was significantly higher proportion of female compared with male CHWs. Perhaps there may be more interest and enrolment into the training program by females compared with males. Yet, this difference in proportion is key, considering need for more male healthcare workers providing services in potentially rough terrain of most developing country rural settings. Features of rough terrain may include difficult access to residents in hard-to-reach settings, increasing insecurity amidst wave of kidnapping and rape, poor rural road networks [14, 15]. Disproportionate gender distribution of CHWs in favor of female, may therefore be impairing their capacity for wider reach of rural residents.

Mean age of respondents was 36.3 years and over two-thirds were within 40 years of age. This suggest relatively younger population rural healthcare workers compared with medical doctors and nurses, who usually require longer years of skilled training. Young population of CHWs may be beneficial in being in the frontline of COVID-19 prevention, considering older age being associated with higher risk of chronic diseases and mortality from the infection [16]. In other words, the relatively young age population of CHWs is favorably suitable for COVID-19 prevention. Younger age also affords the opportunity for longer duration of provision of skilled services before retirement. This potential benefit is also observable in finding most respondents (59.3%) having had 10 or less years duration of practice experience. Yet, this benefit of young age will be fully harnessed, only if their capacities are sufficiently built to provide more effective services [10].

Mean knowledge score of 28.14% ranging from 6.7% to 53.3%, and less than one-tenth having at least 50%, suggest unacceptably low level of knowledge of COVID-19 among CHWs in the study setting. Adequate knowledge is essential for provision of effective health education, including correction of existing myths and misconceptions about COVID-19 pandemic [17]. Unbelief in its existence, as well conspiracy-driven ignorance about the means of disease spread and prevention, requires urgent correction via evidence-based behaviour change communication (BCC) of known facts about COVID-19 [18, 19]. Unfortunately, this low level of knowledge suggests poor capacity for delivery of correct information to rural residents in developing country settings. This is key, considering that providers of BCC must be trustworthy in quality of information provided, in other to enable compliance with effort at ensuring practice of preventive measures [20]. Low level of knowledge may be attributable to novel nature of the virus, as well as potentially much focus on capacity building of other healthcare workers, with relative neglect of CHWs in developing countries [10]. This finding may also suggest lack of effective framework for continuing education among this essential group of grassroots-oriented healthcare professionals.

Regular practice was found to be poor for each of the preventive measures assessed. Poor practice may be attributable to lack of facilities and resources for practice of these measures. This position is supported by finding use of face mask and hand washing as more commonly practiced preventive measure. These resources are perhaps more readily available and cost-effective, compared with goggles, face shield and gloves. Nevertheless, generally poor practice of these preventive measures by CHWs, increases their risk of contracting and spreading COVID-19 infection. Unfortunately, infected CHWs may remain asymptomatic and unaware of their status [21], especially considering abysmally low level of testing in the study area [22]. These HCWs may therefore constitute a nidus for infection and disease spread in rural settings. Coverage of potential area for disease spread may be much wider, considering that Cross River State represents much portion of the easternmost border of Nigeria, with significant land border with neighboring Cameroon in East Africa. Therefore disease acquisition from and spread to East Africa via apparently porous borders, may occur via poor practice of preventive measures by CHWs in Cross River State. Current state of humanitarian crises with massive influx of refugees into the state due to political unrest in neighboring Cameroon, may be contributing significantly to the challenge of providing effective disease control [23].

Another key finding in this study was higher level of knowledge of use of PPE scores, among subjects with regular compared with non-regular practice of hand washing. Though this relationship was not statistically significant, it may have some degree of clinical significance (p=0.07). Also, considering that this relationship was found only for hand washing, it may suggest effect of potentially easier availability of facilities and opportunities for hand hygiene (soap and water), compared with other preventive measures in resource-constrained settings. Yet, proper and consistent hand washing is necessary but not sufficient measure for containment of spread of COVID-19 infection [24]. Therefore, respondents in the study were still at high risk of contracting and spreading the infection to their contacts.

## Conclusion

Community health workers have poor level of knowledge and practice of measures aimed at controlling spread of COVID-19 infection. Their capacity building through training workshops and effective continuing education program are urgently needed. Curriculum for training of CHWs may also need to be reviewed or upgraded, to lay much emphasis on infectious disease prevention and control especially at grassroots level. Further research in other settings using larger sample size is also recommended.

### What is known about this topic

- Due to shortage of health personnel, community health workers are key to health education, especially at grassroots level in rural developing country settings;
- Community health workers also provide basic healthcare services, especially to residents in hard-to-reach rural settings.

### What this study adds

- The knowledge of COVID-19 among community health workers in rural Cross River State was poor;
- There is poor practice of COVID-19 prevention among community health workers in rural Cross River State;
- There is urgent need for capacity building of community health workers towards essential health service provision especially during this critical period of the pandemic.
